# Intronic *NEFH* variant is associated with reduced risk for sporadic ALS and later age of disease onset

**DOI:** 10.1038/s41598-022-18942-x

**Published:** 2022-08-30

**Authors:** Frances Theunissen, Ryan S. Anderton, Frank L. Mastaglia, Ian James, Richard Bedlack, P. Anthony Akkari

**Affiliations:** 1 Perron Institute for Neurological and Translational Science, First floor, RR block, QEII Medical Centre, 8 Verdun St, Nedlands, WA 6009 Australia; 2 Centre for Neuromuscular and Neurological Disorders, University of Western Australia, Nedlands, WA Australia; 3 Centre for Molecular Medicine and Innovative Therapeutics, Murdoch University, Perth, WA Australia; 4 School of Health Sciences and Institute for Health Research, University of Notre Dame Australia, Fremantle, WA Australia; 5 Institute for Immunology and Infectious Diseases, Murdoch University, Perth, WA Australia; 6 Department of Neurology, Duke University, Durham, NC USA

**Keywords:** Motor neuron disease, Neurodegeneration, Risk factors, Genetics research, Genetic association study, Genetic markers

## Abstract

Neurofilament heavy (NEFH) is one of the critical proteins required for the formation of the neuronal cytoskeleton and polymorphisms in *NEFH* are reported as a rare cause of sporadic ALS (sALS). In the current study, a candidate tetranucleotide (TTTA) repeat variant in *NEFH* was selected using an *in-silico* short structural variant (SSV) evaluation algorithm and investigated in two cohorts of North American sALS patients, both separately and combined (Duke cohort n = 138, Coriell cohort n = 333; combined cohort n = 471), compared to a group of healthy controls from the Coriell Institute biobank (n = 496). Stratification according to site of disease onset revealed that the 9 TTTA allele was associated with reduced disease risk, specifically confined to spinal-onset sALS patients in the Duke cohort (*p* = 0.001). Furthermore, carriage of the 10 TTTA allele was associated with a 2.7 year later age of disease onset in the larger combined sALS cohort (*p* = 0.02). These results suggest that the 9 and 10 TTTA motif length may have a protective advantage for potentially lowering the risk of sALS and delaying the age of disease onset, however, these results need to be replicated in larger multicenter and multi-ethnic cohorts.

## Introduction

Neurofilament heavy protein (NEFH) is a type IV intermediate filament. In neurons, assembly of the light, medium and heavy neurofilament proteins is required in a specific stoichiometry (4:2:1 respectively) to form the foundations of the neuronal cytoskeleton^1,2^, to facilitate axonal transport by maintaining axonal diameter, and to direct and position mitochondria along the axon^3,4^. In ALS, one of the histopathological features of disease is the presence of cytoplasmic inclusions containing intermediate filament proteins (light, medium, and heavy) in axonal spheroids and within the cell body of motor neurons^5–8^. The mechanisms behind this increased propensity for aggregation has not yet been fully elucidated, however, there is evidence for hyper-phosphorylation^5,9–11^, dysregulated mRNA expression^12–15^ and altered microRNA levels in ALS patients that can influence the stability of neurofilament transcripts^16,17^ and could contribute to this altered stoichiometry. Typically, the mRNA levels of the smaller neurofilaments (*NEFL* and *INA*) are reported as dysregulated in ALS, whilst *NEFH* mRNA is reported as showing no significant differences between ALS patients and controls^15,18,19^. Alternatively, another report suggests that expression may shift away from *NEFH* to favor the smaller neurofilaments, as an adaptive strategy to reduce energy requirements under conditions of cellular stress during neurodegeneration^20^.

On a genetic level, mutations and polymorphisms in all three NEFH protein domains (head, rod and tail) have been reported to be associated with ALS^21–27^, although studies have typically focused on exonic variants. In particular, large insertions or deletions in the tail region of NEFH have been shown to impact phosphorylation sites critical for its interaction and assembly with other smaller neurofilament proteins^21–23,28^. However, many studies report variants in NEFH as a rare cause of ALS, occurring in only ~ 1% of sporadic cases^21–27^. Despite huge advances in the field of ALS genetics, there is still an urgent need for the characterization of genetic markers that may help to underpin disease heterogeneity and phenotypic variation, particularly in sporadic ALS (sALS) patients^3,29,30^. Therefore, exploring more common short structural variation residing in non-coding regions may help to tag or directly identify genetic factors that may contribute to the role of neurofilaments in ALS disease modification.

In the current study, a candidate intronic TTTA repeat variant in *NEFH* was selected using an *in-silico* short structural variant (SSV) evaluation algorithm^31^ and investigated using PCR, polyacrylamide gel fractionation and Sanger sequencing. A case–control study based on TTTA allele frequencies was performed in two North American cohorts of sALS patients (separately and combined), to determine if TTTA alleles/genotypes are associated with sALS disease risk or have disease-modifying effects.

## Methods

### Study participants and ethics approval from case control cohorts

A combined cohort of 471 Caucasian North American sALS patients and 496 Caucasian North American healthy age-matched controls were used in this study. Participants were diagnosed by board-certified neurologists and met the revised El Escorial World Federation of Neurology criteria for diagnosis of ALS^32^. Routine screening for known ALS genetic mutations was not undertaken. Retrospective genotyping of DNA samples was approved by the Human Research Ethics committee of the University of Western Australia (RA/4/20/5308). DNA and clinical information including age of onset, site of disease onset and survival duration, were collected from 138 sALS patients at Duke ALS clinics in accordance with the Health Insurance Portability and Accountability Act (Pro00040665/323682). In addition, DNA samples from 333 sALS patients and 496 population control samples were obtained from the NINDS Repository, Coriell Institute for Medical Research (New Jersey, USA).

### Polymerase chain reaction

Endpoint PCR reactions were prepared to a final volume of 10 µl, containing; 7.2 µl dH_2_O (Baxter Healthcare, NSW, Australia), 2 µl MyFi reaction buffer (Bioline, NSW, Australia,), 0.05 µl MyFi DNA polymerase (Bioline, NSW, Australia), 0.375 µl forward and reverse primer 5'-GCCTGCTTTGCAGAGCTG-3' and 5'-GGCTCAAGAGATTCTCGTGCC-3', respectively. (Integrated DNA Technologies, Iowa, USA) at 200 ng/µl, and 10 ng DNA. The amplification protocol followed, an initial hold temperature 95 °C for 4 min 30 s and 35 cycles of denaturation at 95 °C for 30 s, annealing 59 °C for 30 s and extension at 72 °C for 1 min. PCR products were then fractionated on a 2% agarose (w/v) gel (Scientifix Pty Ltd, VIC, Australia) in 1 × TAE and stained with red safe nucleic acid stain (iNtRON Biotechnology, Scientifix Pty Ltd, VIC, Australia) prior to imaging using the BioRad Chemidoc™ MP Imaging System.

### Polyacrylamide fractionation and Sanger sequencing

PCR products were fractionated on 8% (w/v) 29:1 polyacrylamide gel (BioRad, CA, USA) in 1 × TBE as previously described^33^. Electrophoresis fragment separation was performed at 100 V for 8 h on the DCode™ Universal Mutation Detection System (BioRad, CA, USA). Gels were stained in 1 × TBE containing SYBR® Gold nucleic acid gel stain (Thermo Fisher Scientific, MA, USA) for 4 min before visualization using a BioRad Chemidoc™ MP Imaging System. PCR products from homozygous samples were purified and Sanger sequenced by the Australian Genome Research Facility (AGRF, Perth Australia). Analysis was conducted using Finch-TV software (version 1.5.0; Geospiza Inc). For high throughput genotyping, a FAM-labelled forward primer was included and the methodology was performed as stated above. PCR products were sent to for capillary separation at the Australian Genome Research Facility (AGRF, Perth Australia). Results were analyzed on peak scanner software (version 1.0; Thermo Fisher Scientific).

### Statistical analysis

Differences in distributions of allelic frequencies were assessed using independent samples Mann–Whitney U or chi-squared tests as appropriate. Case–control genotype/allele associations were assessed using binary logistic regression controlling for patient sex in the covariate adjusted model, with data coded as either the absence (0) or presence (1) of each *NEFH* allele or genotype. Analyses were stratified according to cohort and site of onset, and also combined. Significant associations withstanding Bonferroni correction for multiple comparisons are indicated. Joint significance of genotype/allele associations were assessed via case–control logistic regression. General linear models were used for the association of age of onset with genotype/allele, accounting for patient sex. Kaplan–Meier curves for survival duration were estimated taking into account site of disease onset. Genotypes with low frequency (n < 3) were excluded from statistical analyses. A *p*-value below 0.05 was considered statistically significant. Analyses were carried out in IBM SPSS Statistics version 25.0 (IBM Co., Armonk, NY, USA).

### Ethics approval and consent to participate

Samples from Duke ALS clinic were collected in accordance with the Health Insurance Portability and Accountability Act (Pro00040665/323682). Retrospective genotyping of DNA samples was approved by the Human Research Ethics committee of the University of Western Australia (RA/4/20/5308).

## Results

### Identification of polymorphic TTTA short structural variant in *NEFH*

**Figure 1:**
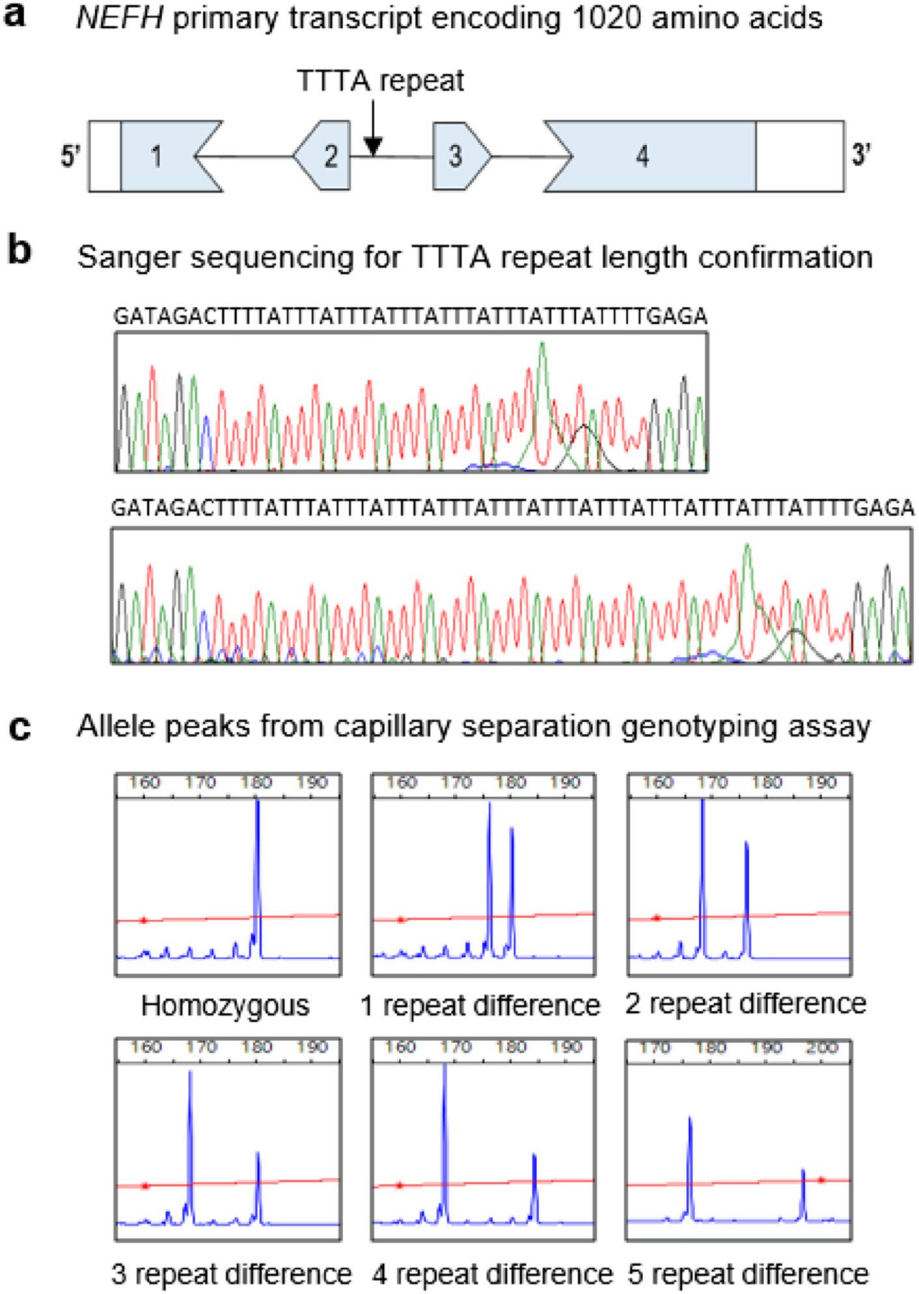
Identification and wet lab validation of polymorphic TTTA structural variant in *NEFH.* (**a**) The primary *NEFH* transcript and location of TTTA repeat 255 bp past exon 2. (**b**) Sanger sequencing demonstrating variable length of the TTTA repeat motif. (**c**) Representation of *NEFH* alleles from capillary separation genotyping assay. The blue peaks depict the fluorescent signal intensity with each peak corresponding to the size of the PCR product capturing the *NEFH* TTTA variant.

A SV evaluation algorithm was used to identify potential polymorphic variants within *NEFH*^31^, with candidate variants scored according to 24 different properties, previously described^34^. The selected TTTA variant was subsequently investigated on public genomic databases NCBI and ensemble genome browser NC_000022.11 and 22: 29483828-29483863 respectively. The recorded entries for this region show a multitude of “rs numbers” logged for this variant, indicating this genetic locus is likely polymorphic and warranted further investigation. The candidate TTTA variant resides 255 bp past exon 2 in the *NEFH* primary transcript encoding 1020 amino acids (Fig. 1a). Polyacrylamide electrophoresis fractionation of *NEFH* PCR products revealed polymorphic alleles with varying numbers of TTTA repeat motifs, and this was confirmed via Sanger sequencing (Fig. 1b). Additionally, capillary fragment separation confirmed variable length TTTA repeat genotypes, with alleles ranging from 6 to 15 TTTA repeats. Representative plots are shown (Fig. 1c), with a single fluorescent signal peak indicating homozygous samples and two signal peaks representing heterozygous samples. As the signal peaks move further apart this indicates a greater base pair difference between the individual *NEFH* alleles.

### Distribution of TTTA alleles

**Figure 2:**
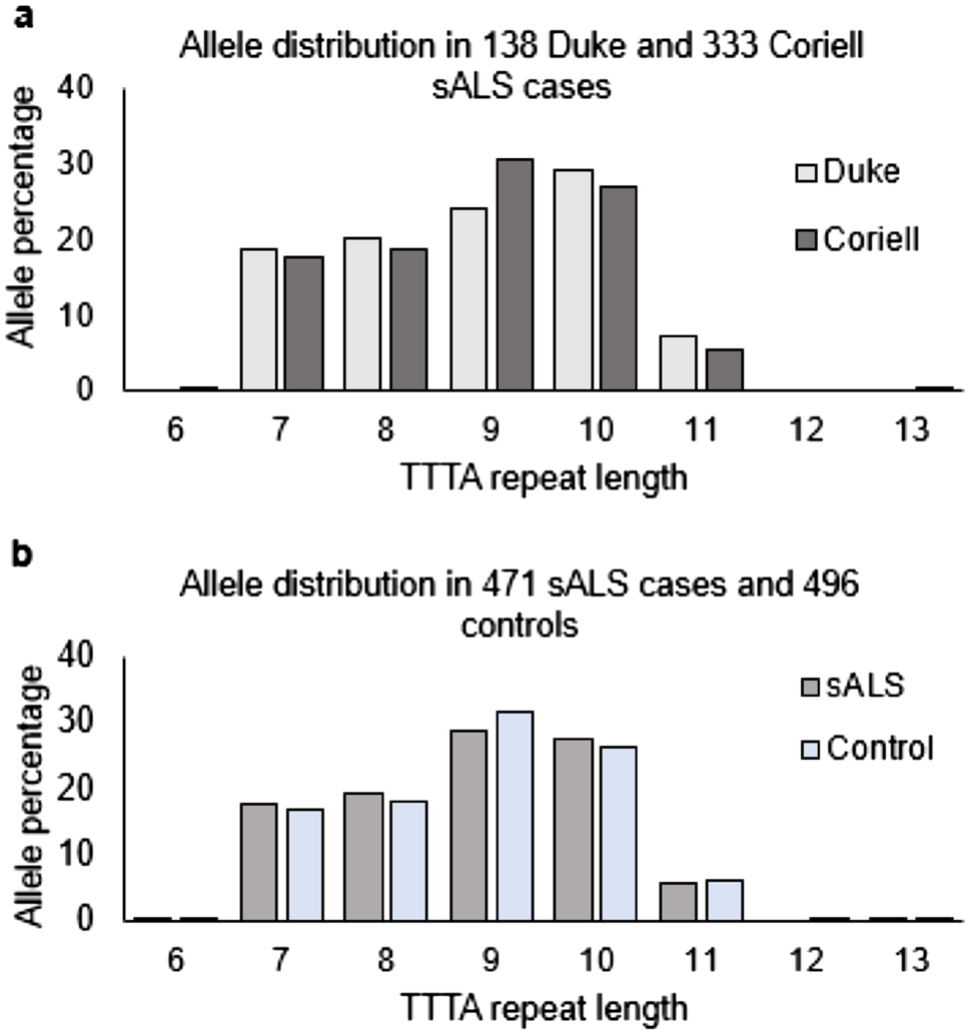
Allele distributions for the *NEFH* TTTA structural variant in two North American Caucasian cohorts (**a**) Comparison of TTTA allele distribution of 138 Duke sALS cases and 333 Coriell sALS cases. (**b**) TTTA allele distribution in the 471 combined sALS cohort compared to 496 controls. Only one sALS patient in the study had a TTTA repeat length of 15 (not shown in figure).

We next investigated the distribution of the TTTA variant in the two sALS cohorts (n = 138 and n = 333) and 496 healthy control cases (cohort demographics can be seen in Table 1). The distribution of allele lengths ranged from 6 to 15 TTTA repeats in both cohorts of sALS patients and in the controls. No significant differences were found in the allele frequency distributions between the Duke and Coriell sALS samples (Fig. 2a). Therefore, both sALS cohorts were analyzed individually and also as a combined cohort (combined allele distribution, Fig. 2b). Along with self-reported ethnicity and country of origin data for each participant, each *NEFH* allele distribution was also compared to Webstr database and was similar to both GTEx (predominantly European self-reported ancestry) and 1000 genomes European allele distributions, providing confidence that the population in this study is reflective of a Caucasian population of European descent. The results are presented as each sALS cohort analyzed individually, in addition to all sALS patients combined, unless stated otherwise.

**Table 1:** Demographics of study participants used for case–control association study.

	Duke cases (n = 138)	Coriell cases (n = 333)	Combined (n = 471)	Controls (n = 496)
Males	76 (55.07)	188 (56.46)	264 (56.05)	256 (51.61)
Age (years)	57.36 (10.91)	54.12 (13.55)	55.05 (12.91)	55.03 (16.76)
Females	62 (44.93)	145 (43.54)	207 (43.94)	240 (48.39)
Age (years)	59.84 (9.46)	58.54 (12.82)	58.93 (11.90)	57.26 (13.47)
Site Spinal	86 (63.24)	249 (76.14)	335 (72.35)	–
Bulbar	50 (36.76)	78 (23.85)	128 (27.65)	–
Age of onset	58.47 (10.32)	56.04 (13.40)	56.75 (12.61)	–
Disease duration (months)	52.7 (39.66)	–	–	–

Note: Displayed as numbers of participants (percentage) or means (standard deviations).

### TTTA variant and sALS phenotype

We first investigated the proportion of ALS subtypes (spinal vs bulbar) within the Duke and Coriell cohorts. The Duke and Coriell cohorts differed in the proportion of spinal and bulbar onset patients (*p* = 0.0066, Table 1). Within the Duke cohort, carriage of the 9 TTTA allele was significantly higher in the bulbar onset cases (*p* = 0.01) however, this effect was not observed in the Coriell cohort (*p* = 0.38). With the significant enrichment of the 9 TTTA allele in bulbar cases within the Duke cohort, we wanted to investigate if the *NEFH* TTTA variant influenced site-specific risk for sALS.

### TTTA variant and sALS disease risk

To explore the association between *NEFH* TTTA repeat length and risk of sALS, a case–control association study was conducted. Binary logistic regression models were used to analyze the carriage of each allele compared to all other alleles in the Duke and Coriell sALS cohorts both separately and combined (Table 2). Comparison of the 9 TTTA allele between Duke sALS cases and controls split by site of disease onset revealed reduced risk specifically for spinal-onset sALS (*p* = 0.001), whilst no effect was seen for bulbar-onset sALS (*p* = 0.60). This effect was not replicated within the Coriell (*p* = 0.38) or the combined cohort (*p* = 0.03). To determine if specific genotypes were contributing to this allelic association we followed up by investigating the genotype carriage in each individual cohort and the combined cohort (Table 3). The 9,10 genotype was associated with reduced risk for sALS by a factor of 0.5 in the Duke cohort, in both the naïve and the covariate-adjusted model for patient sex. However, this was not statistically significant in the Coriell or combined sALS cohort. With both the 9 TTTA allele and 9,10 genotype showing statistical significance in the Duke cohort we wanted to further investigate this association. Within the spinal cases of the Duke cohort, when considering carriage of the 9 TTTA allele, 10 TTTA allele and the 9,10 TTTA genotype; carriage of the 9 TTTA allele remained significant (corrected *p* = 0.001) and was not abrogated by carriage of the 10 TTTA allele or 9,10 TTTA genotype which were both non-significant in the joint model.

**Table 2:** Corrected regression models evaluating the association between *NEFH* TTTA alleles and disease risk in two sporadic ALS cohorts stratified by site of disease onset.

	Duke cases			Coriell cases			Combined		
	n	OR (95% CI)	*p*	n	OR (95% CI)	*p*	n	OR (95% CI)	*p*
7 Spinal	34	1.46 (0.91–2.34)	0.12	82	1.09 (0.79–1.52)	0.59	116	1.18 (0.88–1.58)	0.28
Bulbar	15	0.96 (0.51–1.81)	0.91	20	0.77 (0.45–1.33)	0.35	35	0.84 (0.55–1.30)	0.44
8 Spinal	31	1.12 (0.69–1.8)	0.66	89	1.11 (0.80–1.52)	0.53	120	1.11 (0.83–1.49)	0.47
Bulbar	20	1.33 (0.73–2.42)	0.34	22	0.78 (0.46–1.32)	0.46	42	0.97 (0.64–1.47)	0.90
9 Spinal	28	0.45 (0.28–0.73)	0.001*	121	0.87 (0.64–1.18)	0.38	149	0.74 (0.56–0.97)	0.03
Bulbar	28	1.17 (0.65–2.10)	0.60	43	1.13 (0.71–1.83)	0.61	71	1.15 (0.78–1.70)	0.49
10 Spinal	49	1.52 (0.96–2.42)	0.08	119	1.06 (0.78–1.44)	0.71	168	1.16 (0.88–1.53)	0.30
Bulbar	19	0.71 (0.39–1.29)	0.26	38	1.10 (0.68–1.77)	0.70	57	0.93 (0.63–1.37)	0.71
11 Spinal	10	0.95 (0.47–1.94)	0.89	26	0.85 (0.52–1.38)	0.51	36	0.87 (0.56–1.36)	0.55
Bulbar	8	1.39 (0.62–3.11)	0.42	8	0.83 (0.38–1.81)	0.64	16	1.04 (0.58–1.88)	0.89

Data taken from binary logistic regression models with correction for patient sex.

*P* and OR (95% CI) values are calculated for the comparison to all other alleles.

OR, odds ratio; CI, confidence interval, *p*, statistical significance.

Values in bold with * denote statistical significance after Bonferroni correction *p* < 0.005.

**Table 3:** Corrected regression models evaluating the association between *NEFH* TTTA genotypes and disease risk in two sporadic ALS cohorts.

NEFH TTTA	Duke cohort (n = 138)					Coriell cohort (n = 333)					Combined cohort (n = 471)				
	n	Naïve +		Corrected#		n	Naïve +		Corrected#		n	Naïve +		Corrected#	
		OR (95% CI)	*p*	OR (95% CI)	*p*		OR (95% CI)	*p*	OR (95% CI)	*p*		OR (95% CI)	*p*	OR (95% CI)	*p*
7,7	1	–	–	–	–	11	1.03 (0.47–2.24)	0.95	1.01 (0.46–2.21)	0.97	12	0.78 (0.37–1.68)	0.53	0.78 (0.36–1.67	0.52
7,8	12	1.48 (0.74–2.97)	0.27	1.48 (0.73–2.96)	0.28	21	1.04 (0.59–1.86)	0.88	1.04 (0.59–1.86)	0.89	33	1.17 (0.70–1.95)	0.55	1.17 (0.70–1.95)	0.55
7,9	15	1.17 (0.63–2.15)	0.63	1.18 (0.64–2.19)	0.60	25	0.78 (0.47–1.29)	0.33	0.78 (0.47–1.30)	0.34	40	0.88 (0.57–1.38)	0.59	0.89 (0.57–1.38)	0.61
7,10	20	1.62 (0.92–2.84)	0.09	1.60 (0.91–2.81)	0.10	43	1.42 (0.91–2.20)	0.12	1.40 (0.90–2.18)	0.13	63	1.48 (0.99–2.20)	0.06	1.46 (0.98–2.18)	0.06
7,11	3	0.90 (0.25–3.22)	0.87	0.89 (0.25–3.19)	0.85	5	0.61 (0.22–1.76)	0.37	0.62 (0.22–1.79)	0.38	8	0.70 (0.28–1.72)	0.43	0.70 (0.28–1.73)	0.44
8,8	4	1.03 (0.33–3.17)	0.96	1.02 (0.33–3.14)	0.98	13	1.40 (0.65–3.02)	0.39	1.39 (0.65–3.01)	0.40	17	1.29 (0.63–2.65)	0.49	1.28 (0.62–2.63)	0.50
8,9	14	0.94 (0.51–1.76)	0.86	0.94 (0.50–1.75)	0.84	37	1.05 (0.67–1.63)	0.85	1.03 (0.66–1.61)	0.89	51	1.02 (0.68–1.53)	0.94	1.01 (0.67–1.52)	0.97
8,10	18	1.23 (0.69–2.17)	0.48	1.24 (0.70–2.19)	0.47	27	0.72 (0.45–1.17)	0.19	0.74 (0.45–1.20)	0.22	45	0.87 (0.57–1.31)	0.50	0.88 (0.58–1.33)	0.54
8,11	4	1.03 (0.33–3.13)	0.96	1.02 (0.33–3.16)	0.97	15	1.62 (0.77–3.41)	0.20	1.62 (0.77–3.41)	0.20	19	1.45 (0.72–2.92)	0.30	1.45 (0.72–2.92)	0.30
9,9	10	0.59 (0.29–1.19)	0.14	0.60 (0.30–1.20)	0.15	38	0.97 (0.63–1.50)	0.90	0.98 (0.63–1.51)	0.91	48	0.86 (0.57–1.29)	0.46	0.86 (0.57–1.92)	0.47
9,10	13	0.53 (0.29–0.99)	0.05*	0.52 (0.28–0.98)	0.04*	59	1.10 (0.76–1.60)	0.60	1.10 (0.76–1.59)	0.62	72	0.93 (0.65–1.32)	0.66	0.92(0.65–1.30)	0.62
9,11	5	0.94 (0.35–2.58)	0.91	0.95 (0.35–2.60)	0.93	7	0.54 (0.22–1.30)	0.17	0.54 (0.22–1.30)	0.17	12	0.67 (0.32–1.37)	0.26	0.66 (0.31–1.37)	0.26
10,10	13	1.46 (0.75–2.86)	0.27	1.49 (0.76–2.92)	0.25	22	0.99 (0.57–1.74)	0.98	1.01 (0.58–1.77)	0.96	35	1.13 (0.69–1.84)	0.64	1.15 (0.70–1.88)	0.58
10,11	4	1.03 (0.33–3.17)	0.96	1.01 (0.33–3.12)	0.99	8	0.85 (0.35–2.04)	0.71	0.82 (0.34–1.99)	0.66	12	0.90 (0.41–1.97)	0.79	0.87 (0.40–1.91)	0.74

+ Data taken from binary logistic regression models without correction for covariates.

^#^ Data taken from binary logistic regression models with correction for patient sex.

*P* and OR (95% CI) values are calculated for the comparison to all other genotypes. The following genotypes were excluded form analysis due to low frequency: 6,7; 8,12; 9,15; 10,13; 11,11.

OR, odds ratio; CI, confidence interval, *p*, statistical significance.

Values in bold with * denote statistical significance *p* < 0.05.

### TTTA variant and age of disease onset

To examine the effect of TTTA repeat length on age of disease onset, a general linear model was used. Due to the variability in age of onset data from both Duke and Coriell cohorts, this was investigated in the larger combined sALS cohort to help negate the potential of detecting spurious associations. Within the combined cohort, males had on average a 3.7 year earlier age of onset compared to females (*p* = 0.02). Compared to patients with spinal onset, those with the bulbar onset ALS had a 4.3 year later age of disease onset (*p* = 0.001). In the combined sALS cohort, when analyzed allelically (Table 4), carriage of the 10 TTTA allele was associated with a 2.5 year later age of disease onset in the naïve model (*p* = 0.03). This effect was not abrogated when taking into account the significant covariates including patient sex and site of disease onset, with an estimated mean difference of 2.7 years later age of disease onset following Bonferroni correction (*p* = 0.02). No *NEFH* genotypes passed the statistical significance threshold for being associated with age of disease onset (Table 4).

**Table 4:** Naïve generalized linear model evaluating the association between *NEFH* TTTA alleles/genotypes and age of disease onset in the combined sALS cohort.

NEFH TTTA repeat length	Combined cohort (n = 471)						
	n	Naïve		95% confidence interval		Estimated means	
		β-CoE	*p*	Lower	Upper	Present	Absent
7	157	0.93	0.45	− 1.48	3.34	56.13	57.06
8	165	0.82	0.50	− 1.57	3.20	56.22	57.04
9	224	1.51	0.20	− 0.77	3.78	55.96	57.47
10	227	− 2.50	0.03*	− 4.77	− 0.23	58.05	55.55
11	53	0.06	0.97	− 3.54	3.66	56.70	56.76
***NEFH*** **genotype**							
7,7	12	1.29	0.73	− 5.93	8.51	55.50	56.79
7,8	33	1.92	0.40	− 2.54	6.37	54.97	56.89
7,9	40	2.60	0.21	− 1.48	6.67	54.37	56.97
7,10	63	− 1.90	0.27	− 5.24	1.44	58.40	56.50
7,11	8	3.69	0.41	− 5.11	12.49	53.12	56.82
8,8	17	− 1.42	0.65	− 7.51	4.68	58.12	56.70
8,9	51	3.22	0.08	− 0.43	6.87	53.88	57.10
8,10	45	− 2.80	0.16	− 6.67	1.06	59.29	56.49
8,11	19	1.06	0.72	− 4.72	6.84	55.74	56.80
9,9	48	− 0.48	0.80	− 4.24	3.28	57.19	56.70
9,10	72	− 0.64	0.69	− 3.80	2.53	57.29	56.66
9,11	12	− 0.42	0.91	− 7.64	6.80	57.17	56.74
10,10	35	− 0.64	0.77	− 4.97	3.70	57.34	56.71
10,11	12	− 1.45	0.69	− 8.67	5.77	58.17	56.72

The following genotype were excluded form analysis due to low frequency: 6,7; 8,12; 9,15; 10,13; 11,11. Values in bold with * denote statistical significance *p* < 0.05.

### TTTA repeat alleles and survival duration in the Duke cohort

**Figure 3:**
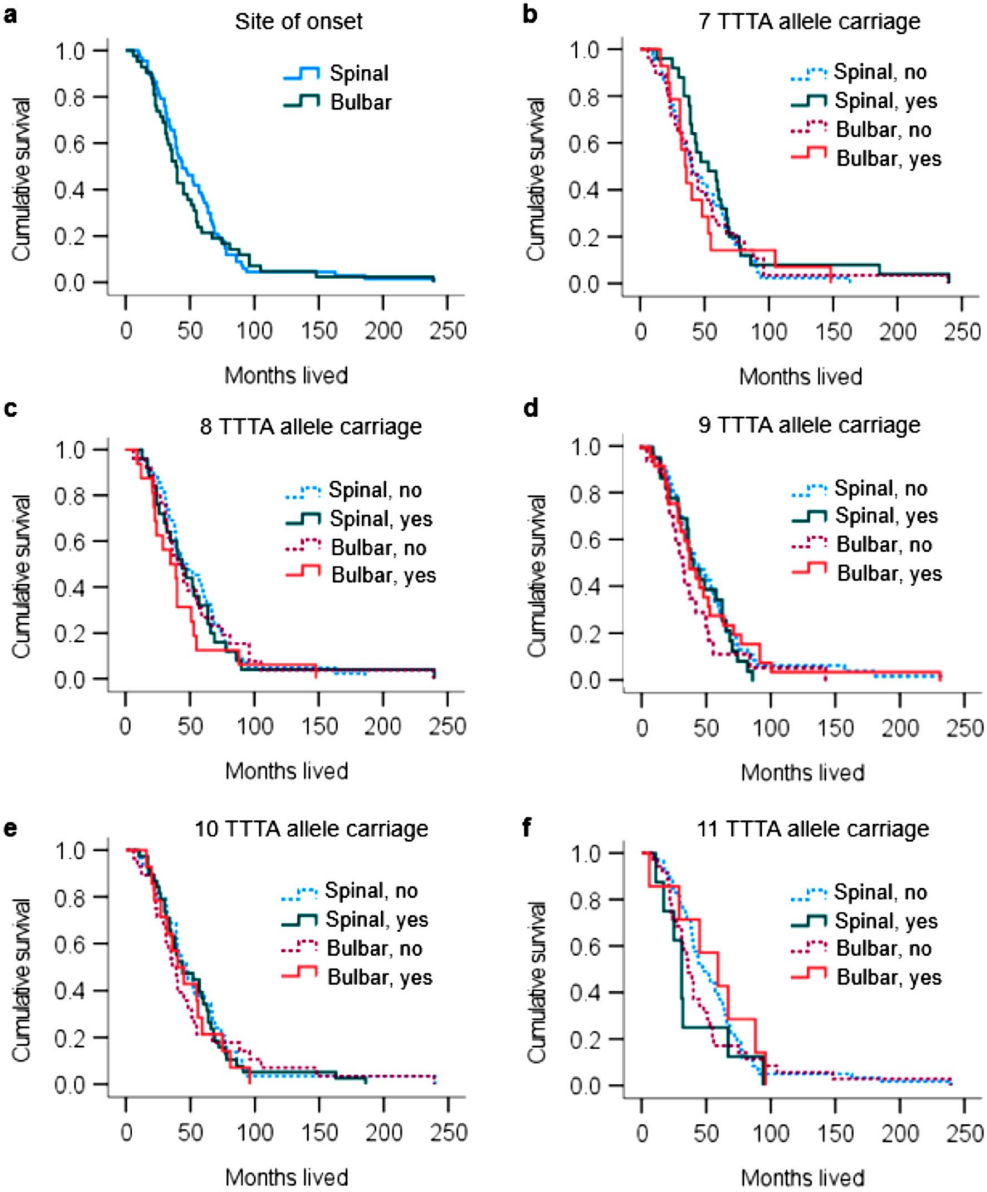
Cumulative survival curves for 138 sALS patients from Duke University (**a**) Survival according to initial site of disease onset. (**b**–**f**) Survival by presence (yes) or absence (no) of *NEFH* TTTA alleles and the initial site of sALS disease onset.

End-point survival data were available only for the 138 sALS cases from the Duke cohort. Within this cohort there was a significantly reduced survival time with increasing age at disease onset (*p* = 0.009). Survival duration was analyzed according to TTTA allele carriage, with site of disease onset taken into consideration. There was no significant difference in end-point survival when stratified by initial site of disease onset (Fig. 3a), nor was there any significant difference in end-point survival for carriage of the 9 or 10 TTTA alleles, or other alleles investigated (Fig. 3b–f).

## Discussion

This study investigated a multi-allelic intronic TTTA structural variant in *NEFH* as a candidate risk factor for sALS, and modifier of age and site of disease onset and survival duration. Within the Duke cohort, carriage of the 9 TTTA allele was significantly enriched in bulbar onset cases. Interestingly, in the Duke cohort the 9 TTTA allele was associated with a substantial reduction in sALS risk by a magnitude of more than one half, but only in spinal onset patients. Within the spinal onset cases, the reduction in risk remained significant after considering carriage of the 10 TTTA allele or the 9,10 genotype, confirming this effect was in fact driven by the 9 TTTA allele. Despite the 9 TTTA association not replicating, it is important to note that the trend for reduced risk in the spinal onset patients was consistent in the Coriell and combined cohorts. On the other hand, there was no association for increased disease risk for any of the *NEFH* TTTA alleles/genotypes in either the Duke or Coriell cohorts, or in the combined sALS cohort.

Following the association with disease risk, *NEFH* alleles/genotypes were investigated as potential modifiers of sALS age of disease onset using general linear modelling. The association with age at disease onset was investigated in the combined sALS cohort to minimize the effect of variability across each separate sALS cohort. Carriage of the 10 TTTA allele was found to be associated with a 2.7 year later mean age at disease onset when accounting for allele carriage, sex and site of disease onset, further supporting the notion of the 9 and 10 TTTA alleles being associated with protective traits in sALS.

Lastly, *NEFH* alleles were investigated as a potential modifier of survival in the Duke cohort. Due to the limited number of patients with end-point survival data, we were only able to analyze the effect of *NEFH* alleles and not genotypes, which we acknowledge as a limitation of this study. We analyzed the carriage of *NEFH* alleles, taking into account the site of disease onset. Although the survival trajectories for spinal and bulbar onset patients did not differ within this cohort, it is important to include this covariate since short structural variants have previously been shown to stratify sALS patient sub-phenotype^33–35^. In the present study, no significant associations were detected between *NEFH* alleles and survival.

A number of possible explanations were considered for the differences in the 9 and 10 TTTA allele associations found in the two sALS cohorts. Firstly, in view of the smaller size of the Duke cohort, the possibility of a spurious association was considered, but in view of the magnitude of the associations and the follow up analyses accounting for the carriage of the 9 TTTA allele, 10 TTTA allele or the 9,10 TTTA genotype, this is considered unlikely. Differences in ethnicity were excluded on the basis of a comparison of self-reported data on ethnicity and country-of-origin from the two cohorts. However, consideration still needs to be given to differences in genetic diversity between the two cohorts, based on the sources of patient samples and geographic patterns of patient recruitment. It is pertinent in this regard that the Duke cohort were from one geographical location (Duke ALS Clinic, Durham North Carolina), whereas cases from the Coriell biobank are multicenter in origin and were recruited from geographically more diverse locations.

Currently, both light and heavy neurofilaments (pNFL and pNFH) are considered among the most promising disease biomarkers for ALS diagnosis and progression, and have been extensively reviewed^5,36,37^. Typically, pNFL levels are considered a more sensitive prognostic marker, displaying a significant correlation between pNFL in the CSF and in serum^38^. On the other hand, pNFH in the CSF does not correlate well with serum pNFH levels but this may be due to the masking of epitopes or post-translational modifications influencing antibody detection^38,39^. Levels of both CSF and serum pNFL and pNFH have been shown to distinguish patients with ALS from controls and other neurodegenerative diseases^38,40^. Various studies have also reported pNFL and pNFH to be correlated with pheno-conversion, clinical measures of disease progression, or the clinical subtype of ALS^39–42^. With this in mind, it would be interesting to investigate if carriage of the *NEFH* 9 TTTA variant correlates with lower levels of pNFH in CSF and/or serum, suggesting a slower rate of degeneration. Particularly, with carriage of the 9 TTTA variant being associated with reduced risk for sALS only in spinal onset patients, it would be interesting to know whether this speaks to a selective vulnerability of different neuronal populations (upper vs lower motor neurons). It is noteworthy that a recent study reported significantly higher serum pNFH concentrations in pyramidal, bulbar and classic ALS phenotypes, compared to flail arm ALS and primary muscular atrophy subtypes in which lower motor neuron involvement was predominant^43^. The study concluded that a positive correlation between pNFH and disease progression suggests that a faster rate of neuronal degeneration may be a determinant of higher serum pNFH levels^43^.

With evidence of NEFH playing a role at both the genetic level and as a prognostic marker for ALS, one must consider the potential impact of previously overlooked short structural variants within the *NEFH* gene in helping to distinguish clinical variability, particularly in sALS. A recent report in 100 sALS patients has stated that 21% of sALS patients carry either pathogenic or likely pathogenic genetic variant in an ALS associated gene, with 13% of patients carrying more than one genetic variant (including variants of unknown significance)^44^. Of note, patients carrying two variants developed disease at a significantly earlier age, with variants in known ALS genes being of potential clinical importance in as many as 42% of sALS patients^44^. This suggests, genetic variation including variations of unknown significance may in fact have a cumulative effect, contributing to disease risk and phenotypic variability between patients. With NEFH having a clear link to ALS disease and progression, future work should explore the impact of short structural variants in *NEFH* and other ALS associated genes as additional markers of disease risk and prognosis.

## Limitations

Importantly, there are several limitations of the present study that should be noted. Firstly, whilst it is known that ~ 10% of sALS patients carry a pathogenic variant in major ALS associated genes^45,46^, the DNA samples that were used in the present study were not screened for such variants. The most common sALS linked variant is the repeat expansion in *C9orf72*^47,48^*,* present in 7% of cases. Although there is phenotypic heterogeneity across carriers of *C9orf72*, the expansion is typically linked with earlier age of onset and with behavioral and cognitive changes, reflective of upper motor neuron involvement^49^. In the present study, the *NEFH* TTTA variant appears to have a protective effect (i.e. reduced disease risk in spinal cases only, and later age of disease onset) and it would therefore be unlikely that the presence of the *C9orf72* repeat expansion would influence the current findings. Once validated, it would be interesting to know if the protective effects of the *NEFH* TTTA variant could reduce the influence of pathogenic variants such as *C9orf72,* and this should be considered in future studies*.* Similarly, the DNA samples were not screened for previously reported *NEFH* exonic variants, considered to be a rare cause of sALS, occurring in ~ 1% of sporadic cases^21–27^ . Secondly, end-point survival data was only available for the smaller Duke sALS cohort. Due to the limited survival data, this could only be analyzed allelically in the Duke cohort and could not be further investigated in the Coriell or larger combined sALS cohort. Finally, the phenotypic data available for the two cohorts examined in this study was limited, therefore, progression measures such as ALSFRS or cognitive status could not be considered in the present study.

## Conclusion

Neurofilament heavy plays a critical role in maintaining the structural integrity of the cytoskeleton within neurons, and when damaged leaks out of the axon into the CSF. Previous genetic studies of *NEFH* have primarily focused on exonic variants which are reported as a rare cause of sALS. The aim of this study was to investigate possible risk and disease-modifying effects of a candidate intronic TTTA variant in *NEFH* within two independent sALS cohorts. Our findings in the Duke cohort point to a substantial reduction in disease risk in carriers of the 9 TTTA allele, specifically in spinal-onset sALS. Collectively, the data suggests that the 9 and 10 TTTA motif lengths may have a protective advantage, potentially lowering both the risk of sALS and promoting a 2.7 year later age at disease onset, as seen in the combined sALS cohort. These results need to be replicated in larger multicenter cohorts with future studies considering more ethnically diverse populations to determine if this risk/disease modifying locus is specific to only Caucasian patients, and to validate this variant as a genetic marker for sALS.

## Data Availability

Data can be made available upon reasonable request. Please contact Professor Anthony Akkari Anthony.akkari@perron.uwa.edu.au.
